# Lyme neuroborreliosis as a cause of sudden sensorineural hearing loss and facial palsy

**DOI:** 10.1002/ccr3.6412

**Published:** 2022-10-11

**Authors:** Letizia Nitro, Barbara Martino, Emanuela Fuccillo, Giovanni Felisati, Alberto Maria Saibene

**Affiliations:** ^1^ Otolaryngology Unit, Department of Health Sciences, Santi Paolo e Carlo Hospital Università degli Studi di Milano Milan Italy

**Keywords:** facial palsy, Lyme disease, meningitis, neuroborreliosis, sensorineural hearing loss

## Abstract

We present a case of sudden sensorineural hearing loss and rapidly progressive facial palsy in a female patient in her 40s with no, apparently, notable past medical or surgical history. Investigations revealed a positive serology for *B. burgdoferi* and the MRI allowed us to identify suggestive signs of Lyme meningitis with multiple cranial nerve involvement. After diagnosis, the patient was treated with intravenous ceftriaxone with a full recovery of sensorineural deafness and facial palsy. This case report highlights the importance of collecting a complete medical history in all cases of facial palsy and sudden hearing loss while presenting an infrequent clinical presentation of early disseminated Lyme disease with neuroborreliosis.

## Graphical abstract

Sudden sensorineural hearing loss and rapidly progressive facial palsy represent an atypical presentation of neuroborreliosis. Complete past medical history collection, prompt diagnosis, and early targeted therapy have a fundamental role in the management of this disease.

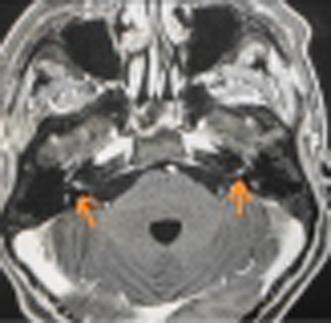

## 1 INTRODUCTION

Sudden sensorineural hearing loss (SSNHL) may result from several causes and, in most cases, it can be ascribed only to an idiopathic etiology. SSNHL is defined as a pure tone audiometry‐proven purely sensorineural hearing loss >30 dB in three consecutive frequencies, developing over 1–3 days.^1^ SSNHL has idiopathic and non‐idiopathic forms. The latter can be due to viral/bacterial infections, vascular disease (with or without compression of vertebral vessels), autoimmune disorders, neoplasms, acoustic trauma, head injuries, Meniere's disease (MD), perilymph fistula, and ototoxicity.^2^ Bilateral SSNHL is rare and a key step in its diagnostic process is excluding a central nervous system involvement by employing adequate imaging.^2^ Our paper aimed to describe how an undiagnosed Lyme disease with neuroborreliosis could induce SSNHL and facial nerve palsy even 1 year later after a *B. burgdorferi* infection.

## 2 CASE REPORT

A woman in her 40s presented at our otolaryngology emergency service (August 2021) reporting a bilateral hearing loss and tinnitus developing over the last 3 days. She also complained of dizziness and painful left head flexion. Despite a significant language barrier, we were able to delineate an uneventful past medical history. She'd been discharged twice in the last 48 h from two other emergency services after being prescribed symptomatic therapy for neck pain, nasal washes, antibiotic ear drops, and oral N‐acetylcysteine, without any other test having been performed other than physical examination. Our otolaryngological examination showed bilateral perforation of the tympanic membrane and residual right eardrum hyperemia, without signs of acute ear infection.

**FIGURE 1:**
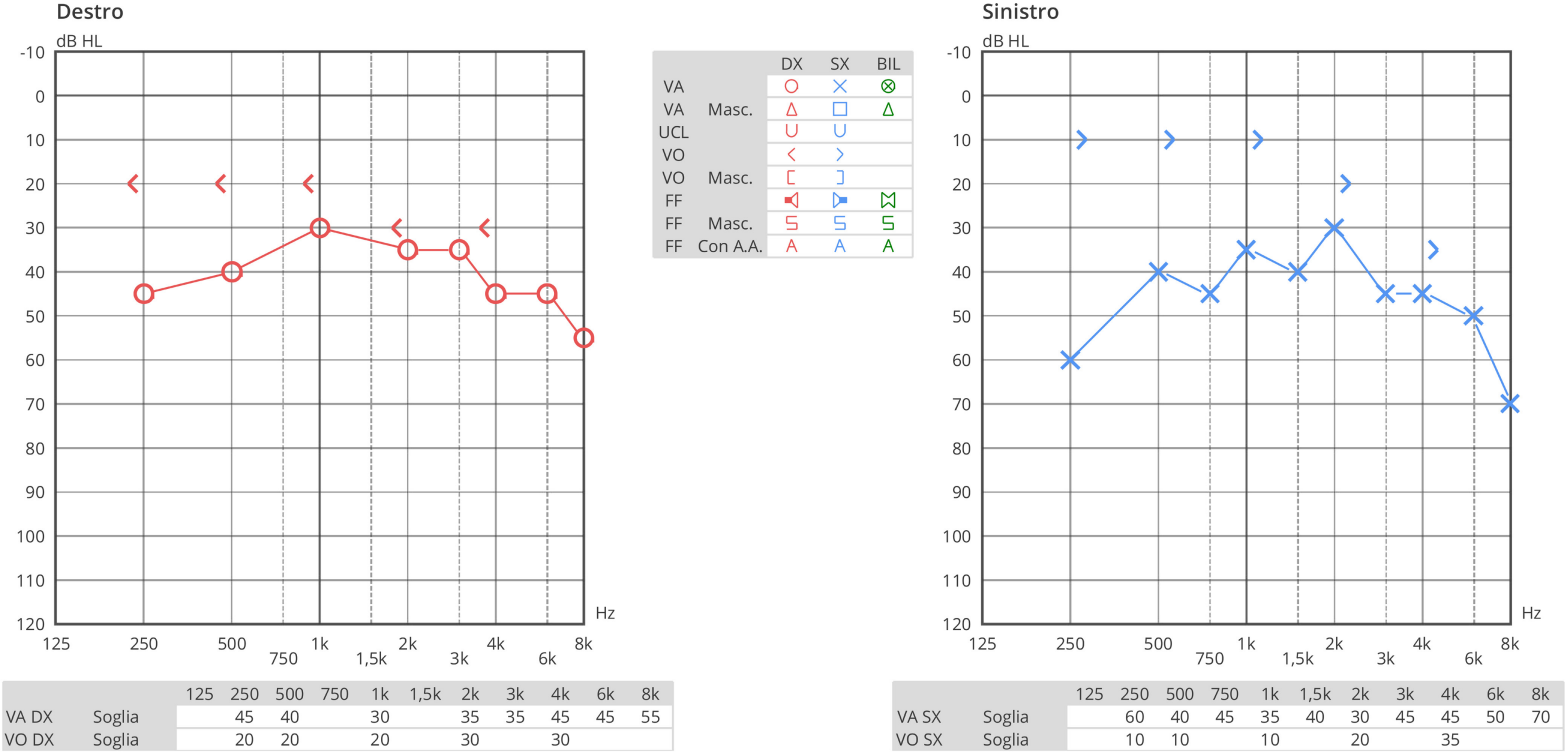
Pre‐treatment pure tone audiometry with evidence of bilateral mixed hearing loss with sensorineural component on acute frequencies at both sides.

**FIGURE 2:**
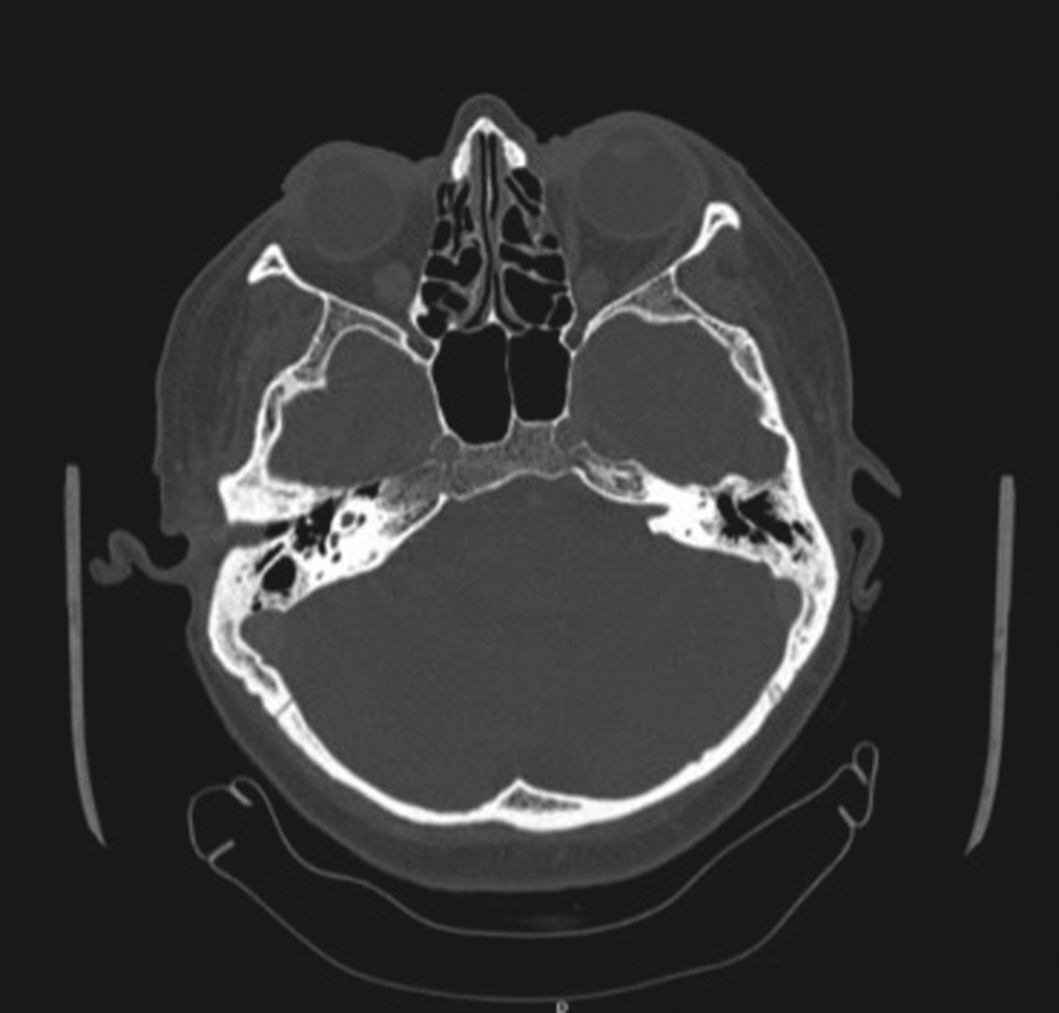
Brain CT scan performed in emergency room, no evidence of acute otitis, mastoiditis, or acute cerebrovascular disease.

**FIGURE 3:**
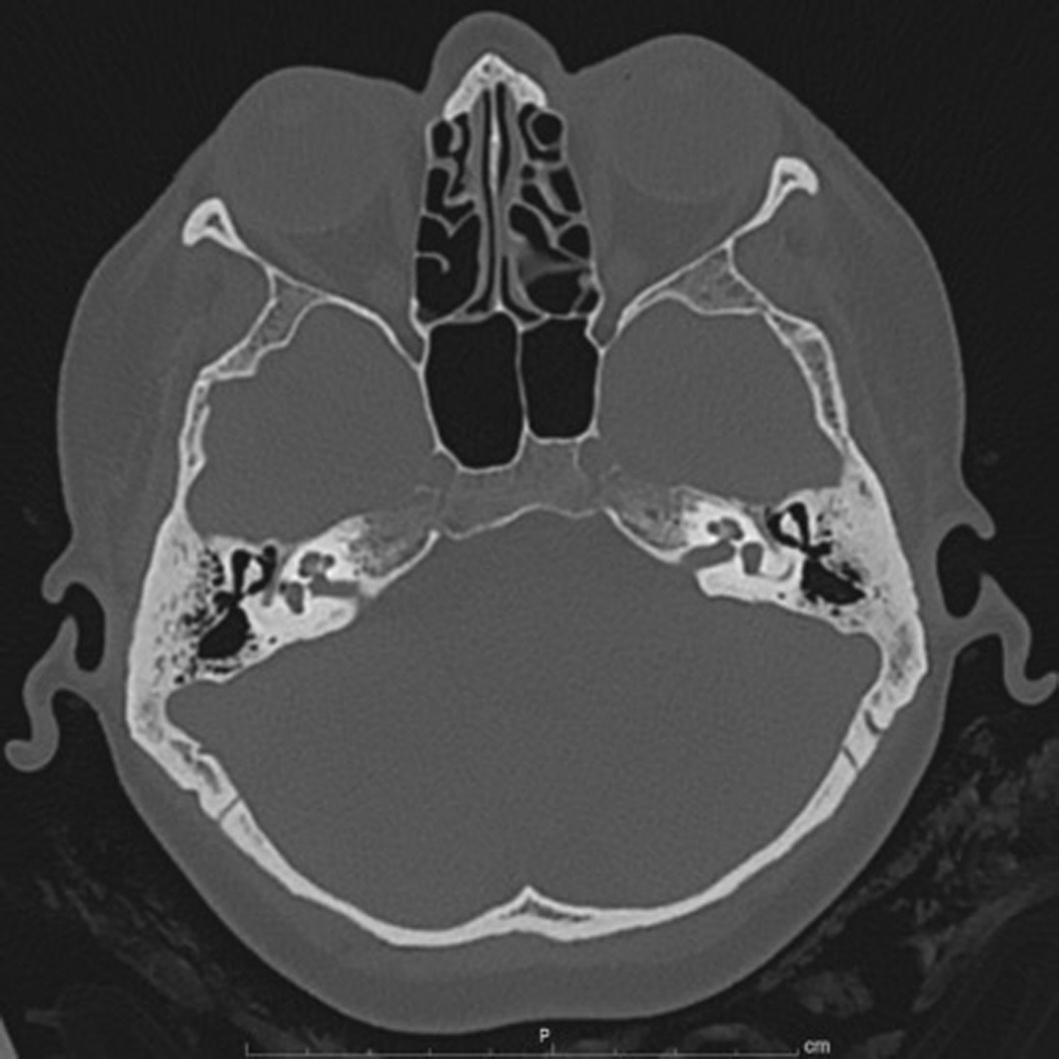
Ear high‐resolution CT scan with no evidence of dehiscence of the tympanic and mastoid tract of the right facial nerve.

**FIGURE 4:**
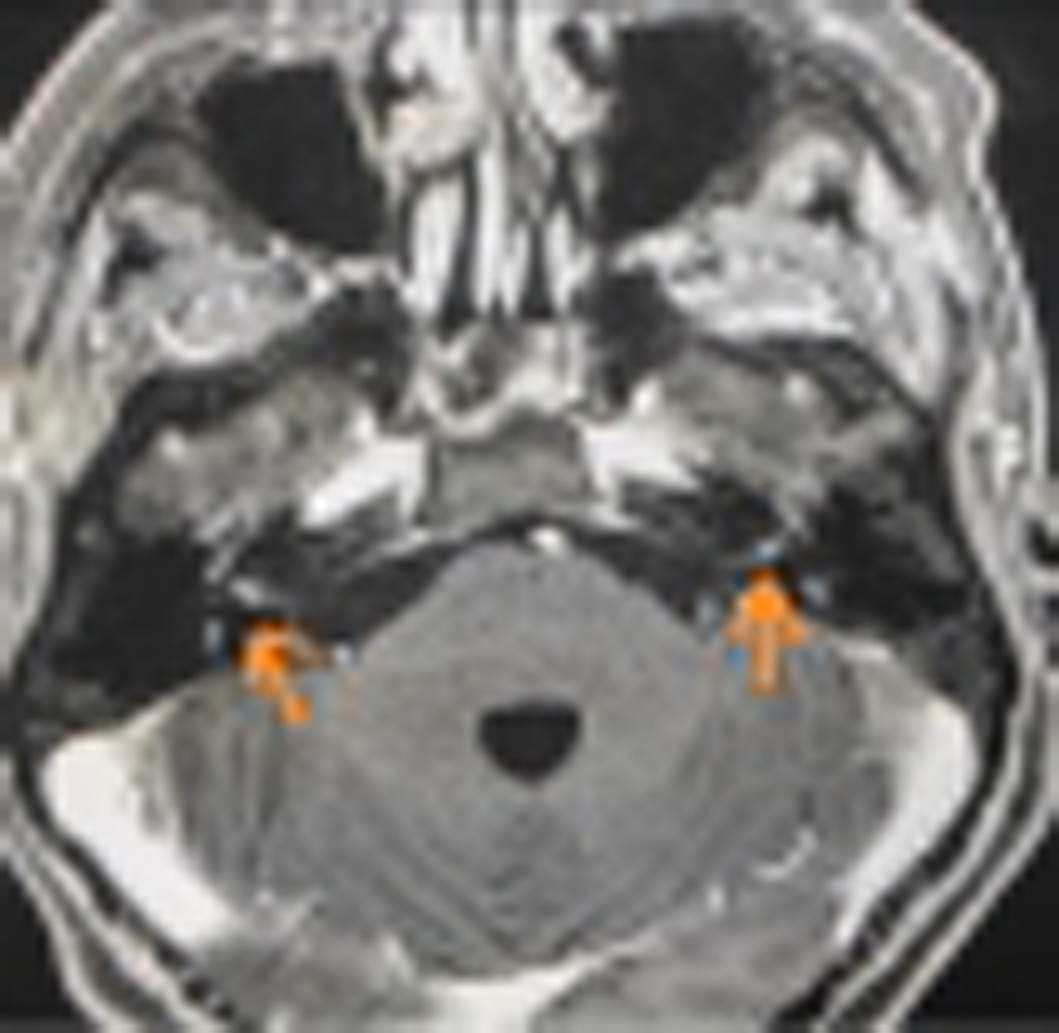
MRI with gadolinium for the study of internal acoustic canal (IAC) and cerebellopontine‐angle cistern. Arrows points the symmetrical and pathological contrast enhancement of the distal portions of the intracanalicular tract of the facial nerves.

The remainder of the otolaryngological examination was unremarkable. Furthermore, the patient did not show any vestibular signs or spontaneous nystagmus upon Frenzel goggles examination. Neck pain prevented performing head shaking test, head impulse test, or complete vestibular bedside examination. The pure tone audiometry bilaterally showed a mixed hearing loss on middle and low frequencies and exclusive sensorineural hearing loss on acute frequencies (see Figure 1). The neurological consult and the head plain computed tomography (CT) were unremarkable. The patient was admitted for overnight watchful waiting and an oral amoxicillin/clavulanate 1 g t.i.d. course was introduced, for suspected otitis, further supported by leukocytosis and elevated blood C reactive protein (CRP). During the night, a new urgent neurological consult was requested following sudden right facial palsy (House – Brackman grade II^3^). As the palsy progressed to a grade III over the next few hours, a new head CT scan was requested and ruled out acute vascular events (see Figure 2). The patient was therefore started on intravenous methylprednisolone 40 mg q.d. and valaciclovir 500 mg b.i.d. and the antibiotic therapy was switched to intravenous ceftriaxone 2 g q.d. The following day, the patient was admitted to our otolaryngology department for further investigations and therapy. Over the next 2 days, neck pain improved, while hearing loss and facial palsy did not show any change. A high‐resolution ear CT scan and a contrast‐enhanced brain magnetic resonance imaging (MRI) were requested, the latter specifically aimed at studying the internal acoustic canal (IAC) and cerebellopontine‐angle cistern. The CT scan (see Figure 3) showed no dehiscence of the tympanic and mastoid tract of the right facial nerve, while the MRI (Figure 4) showed a symmetrical, bilateral, and pathological contrast enhancement of the distal portions of the canalar and intralabyrinthine segments, geniculate ganglion, tympanic tract, and second genu of the facial nerves. During a new and more extensive and detailed medical history collection, the patient reported the removal of a tick from the right external acoustic canal 1 year before. The removal was performed during a trip to southern Italy, and she was not prescribed any further prophylactic therapy.

After a multidisciplinary consultation (otolaryngologist, neurologist, infectious disease specialist, and neuroradiologist) suggested a possible diagnosis of neuroborreliosis with meningitis, specific serological assessments were performed. Western immunoblot for blood *B. burgdoferi* IgG and IgM found evidence only of the latter. Cerebrospinal fluid (CSF) examination following lumbar puncture showed pleocytosis (with lymphocyte predominance) and elevated CRP but failed to identify intrathecal production of anti‐*B. burgdorferi* sensu lato antibodies. Chemokine C‐X‐C motif ligand 13 (CXCL13) count (an aspecific CSF biomarker of Neuroborrelliosis^4^, ^5^) could not be performed for technical laboratory limitations.

**FIGURE 5:**
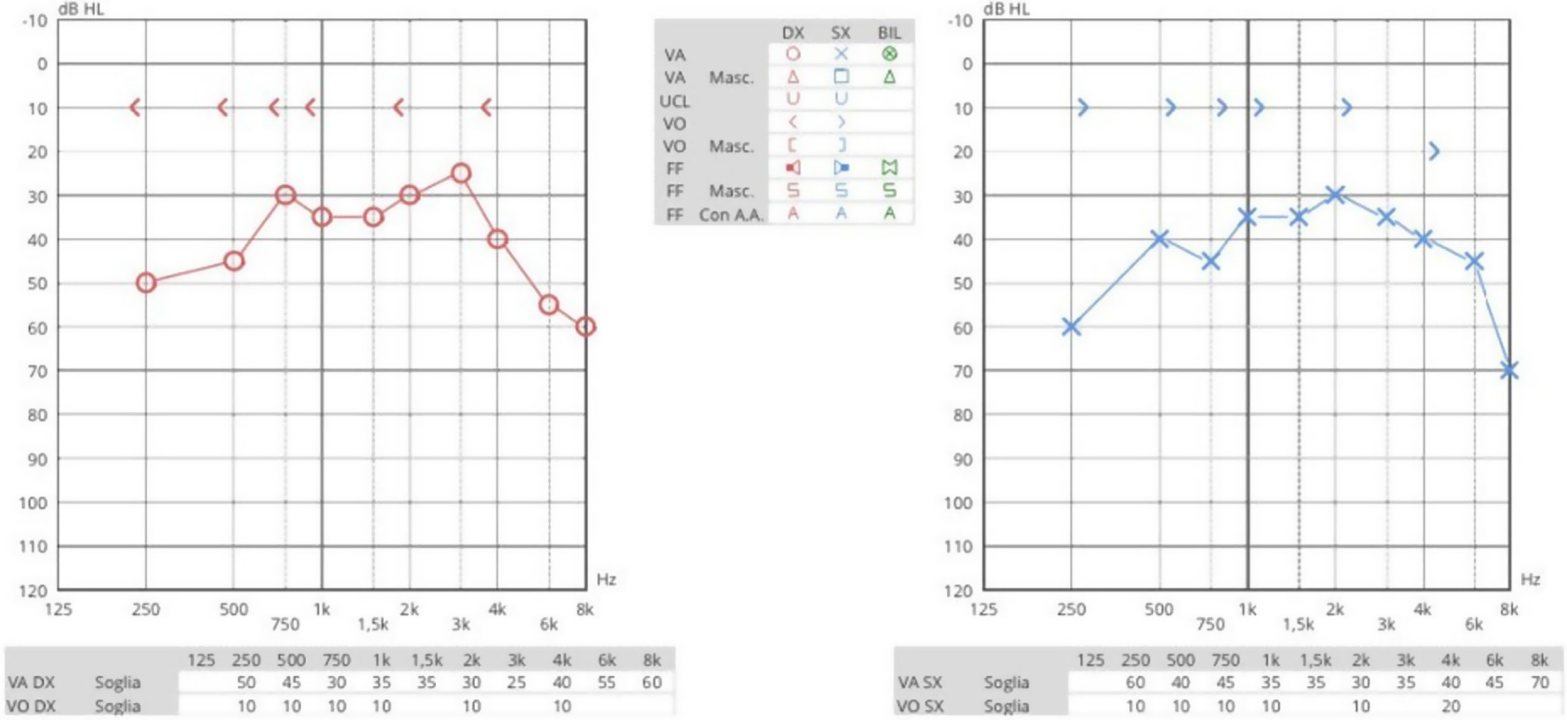
Complete resolution of the sensorineural hearing loss, conductive hearing loss related to bilateral eardrum perforation.

Therefore, according to neuroborreliosis diagnostic criteria, we posed a probable diagnosis.^4^, ^5^, ^6^ The patient was promptly transferred to the neurology department to continue treatment and monitoring. She was discharged after 15 days of hospitalization with complete resolution of the meningeal disease, improved facial palsy, and mixed hearing loss with a bilateral sensorineural component. At the 1‐month follow‐up visit, we found no residual right facial palsy and bilateral conductive hearing loss (see Figure 5), the latter due to the bilateral tympanic membrane perforation.

During this challenging diagnostic process, several differential diagnoses were taken into account. Firstly, mastoiditis due to complicated acute otitis with an inner ear and facial nerve involvement was ruled out due to the lack of clinical, serological, and radiological signs of acute infection. The antibiotic therapy was introduced as prophylaxis due to the rapidly developing neurological signs. The absence of nystagmus and peripheral vestibular syndrome signs allowed us to rule out any vestibular involvement. Indeed, the patient reported only neck pain and stiffness, dizziness, and no head movement‐related vertigo. Peripheral vestibular disorders were further ruled out on the basis of a complete bedside examination with Dix Hallpike, Semont, Rose, and Pagnini‐Mc Clure maneuvers, negative for both sides.

The study of the cerebellopontine angle was mandatory because the association of SSNHL, tinnitus, dizziness, and rapidly progressive facial palsy could be a typical presentation of the acoustic/vestibular schwannoma (although the bilaterality of the hearing loss made this hypothesis less sound).^7^ According to the guidelines,^7^ a contrast‐enhanced brain MRI with T1‐weighted scans study was performed with no evidence of involvement of both cerebellopontine angles and IACs. Analogously, other conditions potentially causing vestibulocochlear nerve were excluded.

Acquired facial palsy could be ascribed to several different causes, among which is worth mentioning infections (e.g., Herpes simplex virus, Varicella‐zoster virus, *B. burgdoferi*, mumps virus, or bacteria causing middle acute otitis), traumata to the temporal bone, malignancies, expansive benignant lesions (e.g., schwannomas) and, rarely, hypertension‐related hemorrhagic events involving the VII cranial nerve.^8^, ^9^, ^10^ Cases in which specific causes cannot be identified are classified as idiopathic (also called Bell's palsy).

The ear CT allows ruling out any fracture of the temporal bone. On the contrary, the contrast‐enhanced MRI allowed for studying the facial nerve and its course (including cerebellopontine angle, internal auditory canal, facial canal along its three portions, geniculate ganglion, and the stylomastoid foramen) and excluding expansive process‐related compression. As detailed above, the only pathological feature the MRI reported was a geniculate ganglion symmetrical enhancement, which allows suspecting neuroborreliosis.^8^ In the literature, even SARS‐CoV‐2 vaccines have been investigated for their possible association with SSNHL due to thrombotic events affecting the internal auditory artery and the labyrinthine artery of the cochlea.^11^ SARS‐CoV‐2 vaccines have been related also to facial palsy events.^12^ Two pathological mechanisms were proposed as explanations: an autoimmune reaction known as a rare variant of a Guillain–Barré Syndrome (BGS) called Bilateral Facial Palsy with paresthesias (BFP)^13^ or idiopathic unilateral Bell paralysis.^12^ However, the patient was uneventfully administered the SARS‐CoV‐2 vaccine 6 months before the events, too long a period to speculate a relationship between our pathological findings and the vaccine.

Otosclerosis, another potential cause of mixed hearing loss, typically shows a progressive conductive hearing loss, which advances to mixed as the cochlear impairment due to the endosteum‐level otosclerotic process could determine modifications of the hearing loss profile.^14^, ^15^, ^16^ In this case report, the sudden onset of hearing loss without CT evidence of otosclerotic cochlear involvement allowed us to rule out this differential diagnosis.

The most challenging feature of this case was identifying a diagnostic connection between SSNHL and rapidly progressive unilateral facial palsy. A suspicious diagnosis of Neuroborreliosis was made thanks to a thorough evaluation of the clinical history and it was confirmed after performing targeted examinations (MRI and serological and CSF evaluation).

According to the current literature on Neuroborrelliosis,^4^, ^5^, ^6^ the patient was treated with intravenous methylprednisolone 40 mg q.d. and intravenous ceftriaxone 2 g q.d. for 14 days.

At the end of the therapy, the patient showed a complete resolution of the meningeal inflammation, facial palsy, and sensorineural hearing loss. After 1 month from the discharge, only a bilateral tympanic membrane perforation remained, as already known at the first examination, and a related bilateral low‐medium conductive hearing loss (Figure 5). The patient is currently well and waiting for a myringoplasty procedure.

## 3 DISCUSSION

According to Sowula et al.,^17^ otolaryngological manifestations of Lyme disease may include several symptoms such as sore throat, otalgia, cervical adenopathy, painless facial nerve palsy, tinnitus, vertigo, and hearing loss. Nevertheless, no literature reports association or correlation between SSNHL and facial palsy except for one case report in which they have been described after a tick bite.^18^ Lyme disease is an *Ixodes species*‐tickborne disease caused by spirochetes belonging to the *B. burgdoferi* sensu lato complex, mainly *B. burgdorferi* sensu stricto, *B*. *afzelii*, and *B*. *garinii*.^2^ According to the European Centre for Disease Prevention and Control,^19^ Italy could be considered an endemic area for some *Ixodes* species, in particular *Ixodes ricinus*. The literature suggests performing a 200 mg single dose intravenous doxycycline prophylaxis within 72 h after prolonged tick attachments.^2^

Lyme neuroborreliosis is reported in 10% of cases of Lyme disease.^20^ Neuroborreliosis causes cranial neuritis mostly affecting the facial nerve or, less frequently, other cranial nerves with ensuing hearing loss, vertigo and/or diplopia.^2^, ^21^ One of the most peculiar features of neuroborreliosis can manifest up to 1 year after a disseminated infection, as in this case report.^2^ It is assumed that the pathogenetic mechanism of the SSNHL in Lyme disease is related both to an immunological reaction and to an angiopathic injury of the cochlea.^17^, ^21^, ^22^ On the contrary, facial nerve involvement seems to be related only to an aspecific inflammatory process.^19^, ^23^ According to the current literature, the diagnosis of neuroborreliosis requires meeting the following criteria: neurologic signs compatible with Lyme neuroborreliosis, CSF pleocytosis (>5 cells × 10^9^ L), intrathecal production of specific antibodies (*B. burgdoferi* IgM and/or IgG).^4^, ^5^ Other aspecific criteria are elevated levels of CRP and CXCL 13 presence in CSF.^2^

Mygland et al. demonstrated that neuroborreliosis can be diagnosed over meeting just two diagnostic criteria.^4^ Although the literature does not delineate a diagnostic role for MRI in Lyme disease, according to Zimmermann et al.,^24^ MRI could be considered a useful tool in the differential diagnosis of facial palsy. In our case report, the evidence of symmetrical and pathological contrast enhancement of the distal tract of the facial nerves, combined with the clinical history, suggested the diagnosis of neuroborreliosis that was subsequently confirmed by serological and CSF assessments. Further studies are required to demonstrate a certain relation between neuroborreliosis, SSNHL and facial palsy and to investigate the pathological mechanisms of disease.

## AUTHOR CONTRIBUTIONS

Letizia Nitro wrote the clinical report. Letizia Nitro and Barbara Martino collected the medical data and critically revised the manuscript. Alberto Maria Saibene, Emanuela Fuccillo, and Giovanni Felisati were involved in drafting the manuscript and helped in the acquisition of data. Giovanni Felisati and Alberto Maria Saibene conceived the publication and revised the manuscript. All authors listed gave final approval of the version to be published and agreed to be accountable for all aspects of the work.

## CONFLICT OF INTEREST

The authors declare that there is no conflict of interest.

## CONSENT

Written informed consent or publishing this report was obtained from the patient in accordance with the journal's patient consent policy.

## Data Availability

Data sharing is not applicable to this article as no datasets were generated or analyzed during the current study.
